# Manufacturing, Characterisation and Mechanical Analysis of Polyacrylonitrile Membranes

**DOI:** 10.3390/polym12102378

**Published:** 2020-10-16

**Authors:** Mertol Tüfekci, Sevgi Güneş Durak, İnci Pir, Türkan Ormancı Acar, Güler Türkoğlu Demirkol, Neşe Tüfekci

**Affiliations:** 1 Department of Mechanical Engineering, Imperial College London, Exhibition Road, London SW7 2AZ, UK; 2 Department of Environmental Engineering, Faculty of Engineering-Architecture, Nevsehir Haci Bektas Veli University, Nevsehir 50300, Turkey; ssevgigunes@gmail.com; 3 Faculty of Mechanical Engineering, Istanbul Technical University, Istanbul 34437, Turkey; pirin@itu.edu.tr; 4 Department of Environmental Engineering, Faculty of Engineering, Istanbul University-Cerrahpasa, Avcilar Kampusu, Istanbul 34320, Turkey; turkan.ormanciacar@istanbul.edu.tr (T.O.A.); gulertde@istanbul.edu.tr (G.T.D.)

**Keywords:** mechanical characterisation, foams, ultrafiltration membrane, finite element method, non-linear deformations

## Abstract

To investigate the effect of polyvinylpyrrolidone (PVP) addition and consequently porosity, two different sets of membranes are manufactured, since PVP is a widely used poring agent which has an impact on the mechanical properties of the membrane material. The first set (PAN 1) includes polyacrylonitrile (PAN) and the necessary solvent while the second set (PAN 2) is made of PAN and PVP. These membranes are put through several characterisation processes including tensile testing. The obtained data are used to model the static behaviour of the membranes with different geometries but similar loading and boundary conditions that represent their operating conditions. This modelling process is undertaken by using the finite element method. The main idea is to investigate how geometry affects the load-carrying capacity of the membranes. Alongside membrane modelling, their materials are modelled with representative elements with hexagonal and rectangular pore arrays (RE) to understand the impact of porosity on the mechanical properties. Exploring the results, the best geometry is found as the elliptic membrane with the aspect ratio 4 and the better RE as the hexagonal array which can predict the elastic properties with an approximate error of 12%.

## 1. Introduction

Water is an essential resource for every life form’s survival. It cannot be replaced by something else and the water resources of nature are not infinite. Thus, treatment and reuse of water is a major issue not for just humans but also for all the existing life on earth. Therefore, engineers and scientists have been constantly trying to maximise the efficiency of the usage of water by developing water treatment techniques. Filtration of water by using membranes is one of the most recently developed water treatment techniques [1,2,3]. On one hand, filtration with a membrane is an efficient technique to treat water; on the other hand, it is quite expensive. This is one of the most important facts, where the idea of this study originates.

Polymeric membranes are commonly preferred for wastewater treatment [4,5]. To tune the permeability of the membrane, pores have to be added to the membrane [6]. Thus, the preparation process is crucial to obtain the desired properties [7,8]. Porous polymeric membranes have different characteristics in comparison to bulk polymeric materials. The first reason is, of course, the size effect of the membrane thickness [9]. The second dominant factor should be given under the concept of porosity [10]. The porosity of the membrane affects the mechanical behaviour since it is directly related to the microstructure [11,12]. The size and the number of pores directly affect the permeability, treatment-related functions as well as the mechanical properties [13]. Polyvinyldenfluoride (PVDF), polyacrylonitrile (PAN), polyetherimide (PEI), and polyethersulfone (PES) are to be enumerated as some of the most widely used polymeric materials for the manufacturing of membranes. Choosing the right polymer is the key point for the treatment of wastewater. Membranes made of PAN, that have high pure water permeability, are typically manufactured in flat sheet geometry by choosing an appropriate component of the casting solution and the conditions for fabrication. However, with its wide tolerance range to organic solvents, PAN membranes are used abundantly in the pervaporation process, industrial wastewater treatment, and the manufacturing of composite membrane substrates [14]. To form pores in the membranes, some agents are added in the main polymer [15]. Adding PVP into PAN provides checking over the properties of polymeric membranes, especially thermodynamically and kinetic-wise [16,17]. Addition of PVP causes thermodynamic instability and phase separation is to be observed [18]. Consequently, the blending of materials, which become soluble, is delayed and the polymer solution with a level of considerably significant viscosity goes through the process of phase separation [19].

The majority of studies are concerned with the manufacturing and characterisation of membranes [20,21,22,23,24,25,26]. As a result of these works, it is pointed out that many properties, including the mechanical properties, of membranes, can be tailored by controlling the micro-nano structure of the materials [27,28,29,30,31]. However, the prediction of the mechanical properties of polymeric materials with good precision, as can be achieved with metals, is hard. This is because of the complicated and amorphous micro-nano structure of polymers [32]. It is well known that a material’s structure affects the mechanical properties. However, in the case of polymers, the impact is much more significant [33]. The importance of the material structure has attracted much attention in the world of science and research. The synthesis of polymeric materials is the main process by which the structure is significantly formed [34,35]. Thus, polymeric materials are to be tailored by taking advantage of this [36,37].

As well as the prediction of mechanical properties, modelling of the polymeric materials is another problem that has to be handled carefully. One of the deepest rooted approaches is the linear elastic behaviour, which is usually not representative of polymers [38]. Polymers have a nonlinear nature in terms of mechanical behaviour, namely deformations, stress-strain relationships. They exhibit large deformations under relatively small loads compared to metals, which makes it possible to neglect the thermal changes caused by loading and deformation [39,40,41]. This is called the theory of hyperelasticity. However, this theory does not cover the time-dependent behaviour of polymeric nature [42,43]. In statically loaded cases, time-dependent nature is dominated by relaxation. This behaviour is represented by the viscoelastic theory [44]. The dynamic behaviour is affected by this time-dependent nature as well [45].

Alongside all these factors, the size effect is to be considered for these types of materials as well [46,47]. Combining the macro and nanoscale behaviour of the materials is also another important research field [48,49,50]. A relatively new method has emerged considering the molecular behaviour of materials, namely molecular dynamics [51,52].

Polymeric foams have an even more complicated structure and mechanical behaviour. Their porous microstructure coupled with the natural nanostructure of polymers causes their very complex and hard to predict behaviour [33,53]. One of the most common and practical methods to conduct the modelling for polymeric foam is the finite element method [54,55,56]. Representative volume element analysis is also a widely used method [57]. The representative element (RE) methods allow researchers to obtain a better insight into the influence of the localised behaviour on the global behaviour [58,59]. The major problem to face in the analysis of REs is the determination of their size and the size and the distribution of the inhomogeneities [60,61]. To determine these parameters, stochastic approaches are common in the literature, as well as regular distribution assumptions. The stochastic approaches provide better accuracy since they require multiple repetitive stages of computations [62,63]. Hence, they require more computational resources and time. The regular distribution assumption of inhomogeneities enables the direct determination of the size of the RE and the inhomogeneity parameters. Therefore, the calculations to obtain these parameters are eliminated and no repetition is required unless a change in parameters is desired.

Alongside polymeric foams, polymers with reinforcements, namely composites, are also used in water treatment as well as many other engineering applications. The reinforcements can be in different forms such as particle, platelet/sheet or fibre, and materials like graphene, carbon nanotubes, and clay. These reinforcements affect mechanical properties significantly as well as the treatment performance of the membranes [31,64,65].

As one of the major problems of membrane systems, mechanical behaviour and strength of membranes are handled in this study. Some studies aim to improve the mechanical properties alongside the strength of membranes by adding additives and/or applying various chemical processes to their manufacturing recipe [66]. Meanwhile, apart from these techniques, this study introduces a new perspective and deals with the membrane structure and geometry to make the most of the existing membrane strength without adding any complications to the preparation processes.

The membrane shape is strongly influenced by the pressure applied. Thus, the geometry is found to be one of the most dominant effects that are influence on the maximum load that can be carried by the membrane. Besides this, as mentioned before, the porosity and the pore distribution are also to be enumerated among the dominant structural parameters [67,68]. These parameters are not only influential on mechanical strength but also on the operating life-time of the membranes. In light of all these, this work aims to predict the mechanical behaviour of membranes of elastic characteristics by using a membrane which is manufactured and experimentally characterised within the scope of this study.

More specifically, this study deals with the numerical modelling of the mechanical behaviour of porous polymeric membranes relying on the data acquired from the experiments that are conducted on the manufactured membranes. As mentioned earlier, a significant portion of the research in the literature only prepares and characterises the membranes, whereas too few of these studies include modelling works with the results they obtain from the characterisation process [69]. These membranes are designed to treat fruit juice wastewater in a “Submerged Membrane Bioreactor” (MBR) [17]. Material characterisation is done by performing experiments [70]. To obtain material behaviour, the uniaxial tensile test is conducted [38,41]. The outcome of this experimental process feeds the numerical study as the output data are used as input for the finite element analysis of the membranes that are manufactured using these materials. A key point of this study is to include the large deformation, which is a type of geometric nonlinearity [71,72]. These analyses aim to find better geometry to design a membrane under the same loading and boundary conditions which are representative of the actual operating conditions of wastewater treatment membranes. To compare the geometries, the maximum deformations, von Mises stress, reaction forces, and the equivalent strains are used as criteria. As one of the most important geometric properties, the aspect ratio is considered in the scope of this study. Moreover, the effects of porosity are investigated numerically using two different types of RE with different pore distributions. This aims to explain how porosity influences the change in stiffness and strength based on the porosity and the pore size. The representation capabilities of the two types of RE are compared and discussed. The RE sort with better representation capability is taken for further analysis. The porosity values are changed between 5% and 70% and the RE models are built. The changes in mechanical behaviour and mechanical parameters depending on the porosity values are investigated. The methodology presented in this study significantly reduces the number of experiments necessary to develop a better membrane system as well as the cost and time to be spent on experiments.

## 2. Materials and Methods

This section describes the processes by which the membranes are manufactured and characterised. Two types of membrane materials are prepared for membrane manufacturing. One is with PVP addition and the other one is without. The characterisation covered scanning electron microscopy (SEM, Thermo Fisher Scientific, Inc, Waltham, Massachusetts, USA), atomic force microscopy (AFM, Courtesy Of Digital Instruments, Santa Barbara, California, USA), Fourier transform infrared (FTIR, Spectrum 100, Perkin Elmer, Inc., Waltham, Massachusetts, USA), permeability tests, mechanical uniaxial tensile test, and determination of other various properties.

### 2.1. Preparation of Dope Solutions

PAN (CAS Number 25014-41-9) and PVP (CAS Number 9003-39-8) polymers are purchased from Sigma Aldrich, St. Louis, MI, United States. N-Methyl-2-Pyrrolidone (NMP) (8060722500) solvent is purchased from Merck Kenilworth, New Jersey, United States.

PAN polymer is added to NMP solvent and stirred at 110 rpm at 40 °C until the mixture became homogeneous. This procedure is used for the dope solution of PAN 1 membrane. To prepare a dope solution of PAN 2 membrane, firstly, PVP is added into the NMP solvent. After PVP is dissolved, PAN polymer is added into the solution and stirred under the same conditions. The contents of PAN 1 and PAN 2 membranes are summarised in Table 1.

### 2.2. Membrane Forming

Homogenous dope solutions are cast onto glass plates, making sure that no air bubbles remained in the casting solutions. To form pores in the membrane, a dope solution, that is cast to the glass plate, is kept for 10 s in the air. After waiting, the glass plate is submerged in distilled water for coagulation. The casting solution is cut with a knife with a gab of 200 µm. Fabricated membranes are stocked up in wet condition/in water and in the cold room before experiments.

### 2.3. Characterisation of Membranes

The porosity of PAN 1 and PAN 2 is determined by using the dry-wet method. This method uses the following expression to calculate the porosity of the membranes:

(1) $$\mathrm{Porosity}=\frac{w_{2}-w_{1}}{v\times d_{\mathrm{water}}},$$

where $w_{1}$ and $w_{2}$ are the weights of the dry and wet membranes, respectively, v is the membrane volume and $d_{\mathrm{water}}$ stands for the density of the distilled water at room temperature. Water content is determined in percentage by applying the dry wet method. The mathematical formula for calculating the water content is given below:

(2) $$\mathrm{Water}\;\mathrm{Content}=\frac{m_{1}-m_{2}}{m_{1}},$$

where $m_{1}$ is the membrane mass after being dipped in distilled water for 24 h and $m_{2}$ is the membrane mass after being kept in the incubator at 45 °C for 48 h. Moreover, surface polarity and contact angle are measured for membrane characterisation. Attension T200 Theta is used for these measurements and at room temperature, a 5 µL droplet is sent to the surface of the membrane. Three repetitions are conducted for each set of experiments, and to obtain the final result, the results of the repeated tests are averaged.

Viscosity is one of the basic properties of the material and it is also important for membrane characterisation. Therefore, it has to be determined to understand material behaviour. Measurements of membrane viscosities are conducted using a Brookfield DV-E Viscometer with a shear rate of 100 s^−1^ at 20 °C for two minutes utilising a cone/plate geometry.

Another parameter for obtaining a deeper understanding of the material is the pure water flux. This is more about the specific behaviour of the membrane while the water is being treated. The measurement of pure water flux (PWF) is performed at 20 °C and under 600 mbar pressure. Following this, the pure water flux is computed using the equation below:

(3) $$J=\frac{Q}{A\Delta t},$$

where Δt stands for the sampling time, J is the PWF, A is membrane surface area and Q is the quantity of permeate collected at the steady-state of the membrane.

PAN 1 and PAN 2 membranes are analysed with a scanning electron microscope (SEM) using FEI Quanta 450 FEG-EDS. Moreover, AFM analysis is performed using a device from Digital Instruments, and FTIR analysis is performed using a Perkin ELMER Spectrum 100 FTIR instrument for surface and structure characterisation analysis.

As one of the most commonly used experimental methods, tensile testing is conducted on the material samples to understand the mechanical behaviour of the materials. Since this test is performed from zero stress to failure, both the elastic and plastic behaviour of the materials could be characterised under quasi-static loads.

To apply this method, the ASTM 638 standard method is employed for three specimens from each material using a dynamic mechanical analyser (DMA—Seiko Chiba, Chiba, Japan, SII Nanotechnology DMS6100 200 Hz) [73]. The dimensions of the rectangular specimens can be described as 15 mm by 3 mm with a thickness of 1 mm. First, to reduce the effect of looseness, a stabilisation force of 1 N is applied for 5 s on the specimen. Following this, a constant strain rate of 20 µm/min is applied to the material specimens and the reaction forces at the supports are measured. The stress and strain calculations are performed based on these data acquired using true stress and strain assumptions. Such a small strain rate is applied to estimate the quasistatic behaviour of the material, as mentioned earlier. Alongside the strain rate, the temperature also affects the behaviour of polymeric materials; therefore, the ambient temperature is kept constant and noted as 25 °C. Finally, the Poisson ratio is also determined using the appropriate equipment.

## 3. Numerical Study

The numerical part of this study consisted of building a finite element model of the membranes with different geometries and analysing the stress distribution in the REs with the same numerical method, which is carried out using the commercial software packages SolidWorks 2018 (Dassault Systèmes, Vélizy-Villacoublay, France) and Abaqus 2016 (Dassault Systèmes, Vélizy-Villacoublay, France). The first part of the numerical study is a direct analysis of the geometry dependency of the characterised membranes and the second part is the investigation of the porosity dependence of the material PAN.

### 3.1. Modelling of Membranes

Here, the aim is to investigate how the shape affects the stress distribution throughout the membrane as well as the maximum stress and the effect of the material properties on the behaviour of each shape. Material properties are represented with an elastoplastic material with a linear elastic region in this numerical model, which is built using the data obtained from the uniaxial tensile testing.

To eliminate other parameters, the boundary conditions, total surface area, and the applied pressure are taken to be the same for each membrane with different geometries. The membranes are fixed at outer boundaries. Having the same surface area of 49 mm^2^ and the pressure load led to the same total load on each membrane. The pressure load is uniform all over the membrane and stayed normal to the surface as the membrane deformed from increment to increment. The magnitude of the pressure load is taken as 5 kPa to keep the deformed membranes within their elastic regions rather than aiming to be realistic. The loading type and the boundary conditions are representative of the real operating conditions; however, the main purpose is not to predict the accurate stresses under certain loads since PAN material is not used in membranes without reinforcements such as fibres and/or supporting layers. Membranes are modelled as plates using a quadratic shell element which also includes the membrane behaviour. It is also important to note that the membrane material is considered as homogenous isotropic material. Considering the geometries, the bending stiffnesses are expected to be extremely low, so this will make the membrane behaviour dominant for these cases. To show that this assumption is valid, membrane and bending stresses are calculated together and the resultant stresses are used while calculating the equivalent stresses with the criterion of von Mises. However, von Mises is not an appropriate failure theory for such polymers; it is used as an acceptable principal stress combination to compare the stress magnitudes among the selected geometries. Displacement and the equivalent strain are also used as criteria of comparison.

### 3.2. Modelling of Representative Elements

Modelling of the RE deals with the localised and small-scale behaviour of the porous material of the membranes. The main objective is to understand how porosity influences the stiffness and the strength of the membrane material.

To investigate the effects of the porosity, two sorts of RE with different regular distributions of uniformly sized circular pores are generated. The first type of RE is the hexagonal distribution, which is called RE 1, and the second one is the rectangular distribution, which is called RE 2. As the first step, two REs are generated from each type of RE with porosity values for PAN 1 and PAN 2, which are marked with a and b, respectively.

Membranes displayed a biaxial plane stress state. Therefore, the REs are two-dimensional and they are prepared under plane stress assumption. The four straight edges of the REs are subjected to periodic boundary conditions and a uniaxial tensile strain of 0.1%.

The elastic properties of both of the RE types are calibrated based on PAN 1 since it is the less modified version among the two materials manufactured. Using this, the elastic modulus value for fully bulk material is estimated by employing mean-field homogenisation. This is taken as the elastic modulus for the material used in RE calculations. Finally, both types of RE are tested and the one with the better representation capability is used to investigate the effects of porosity on the strength and stiffness of the material. The porosity values are chosen to start from 5% and increase up to 70% with 5% increments. Their finite element models are built under the same boundary conditions and assumptions.

## 4. Results and Discussion

### 4.1. Material Characterisation

The material properties determined through the experimental processes are presented in Table 2. As the first observations, it can be claimed that the PWF increased with increasing porosity for obvious reasons.

**Figure 1:**
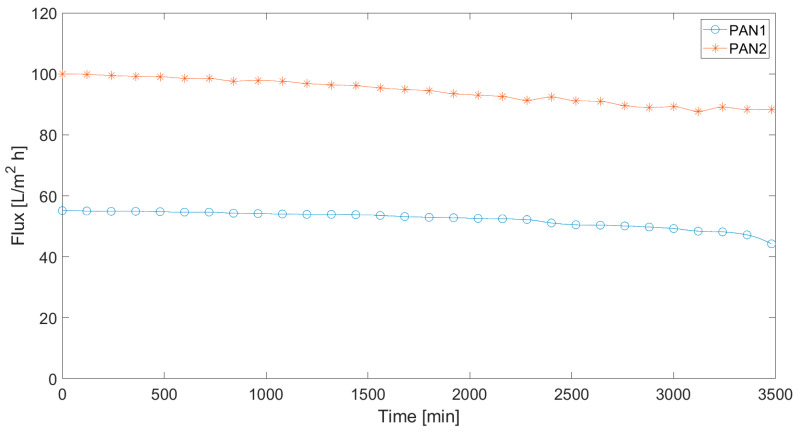
Time dependency of the pure water flux passing through the membranes.

The time-dependent values of pure water flux through the membranes are displayed in Figure 1. The pure water flux is stable over time. However, a small decrease after 2500 min is still observed. Because of the applied pressure, the pores of the membrane are compacted and a small decrease can be assumed as normal behaviour [74]. Compared to PAN 2 membranes, less flux is observed in pure water filtration from PAN 1 membranes. This is because of the lower porosity of the PAN 1 membrane. Moreover, when the contact angles are examined, PAN 2 provided higher filtration compared to the PAN 1 membrane due to its higher hydrophobicity and low water holding capacity [75,76,77,78].

**Figure 2:**
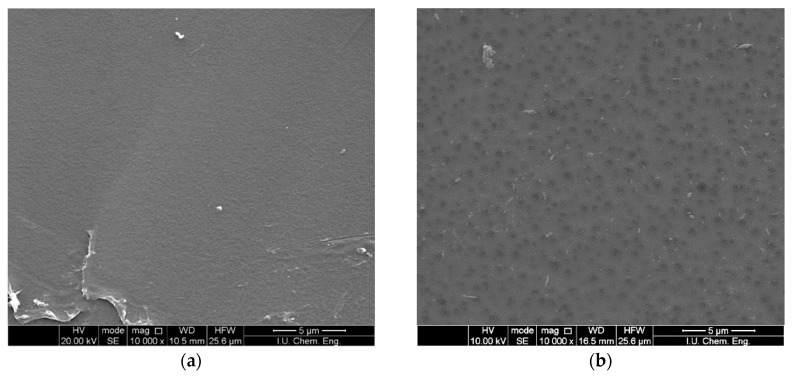
SEM images of membranes (5000×) (**a**) PAN 1; (**b**) PAN 2.

Table 1 and PAN 2 membranes are given in Figure 2, where it can be seen that the pore size of the PAN 2 membrane is higher than the PAN 1 membrane.

**Figure 3:**
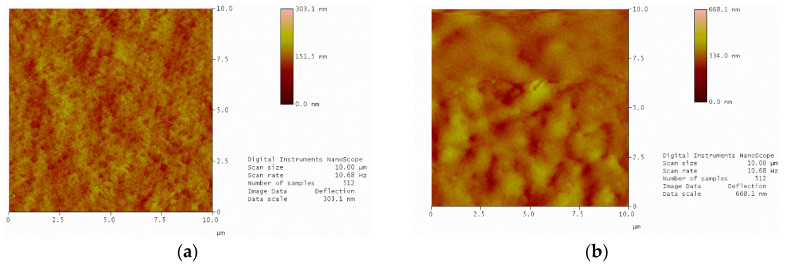
AFM images of membranes (**a**) PAN 1; (**b**) PAN 2.

Figure 3 displays the results of the AFM analysis. Addition of PVP increased the surface roughness, which led to stress concentration in microscales and initiated failure more easily compared to smoother surfaces. This also had a significant impact on the stiffness as well as the strength of the materials. Therefore, it can be claimed that this correlation is valid for the surface roughness and the mechanical properties of PAN 1 and PAN 2.

**Figure 4:**
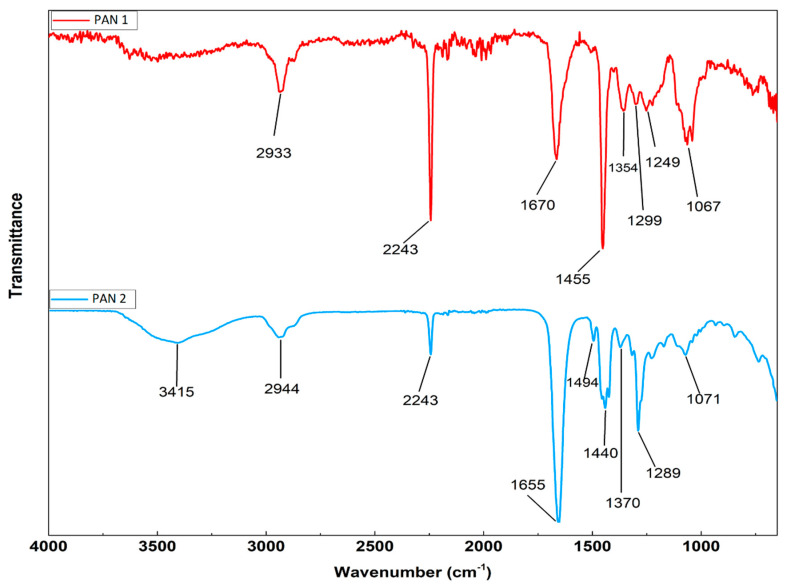
Results of FTIR analysis of PAN 1 and PAN 2 membranes.

When the FTIR results of PAN 1 and PAN 2 membranes are compared, a peak is observed at 3415 cm^−1^ in the spectrum of PAN 2, probably due to the presence of absorbed water. The peak at 2933 cm^−1^ in the PAN 1 spectrum shifts 2944 cm^−1^ in the PAN 2 spectrum. The peak at 2935 cm^−1^ is assigned to –CH_2_- and the bands of 2240 cm^−1^ and 1450 cm^−1^ indicate –C≡N groups [79]. The peak at 1655 cm^−1^ can be attributed to the C=O group in the amide structure [79,80] and the change in the band in the region of 1690–1630 cm^−1^ corresponds to C=O groups of the PVP [81]. The change in the transmittance of this band indicates the effect of PVP on the PAN structure. The PVP has the characteristic peaks at 1290 cm^−1^, 1660 cm^−1^, and 1463 cm^−1^ which are assigned to stretching vibration of C–N, C=O and CH_2_ bonds, respectively, and the stretching vibrations of C–N bonds from the PVP group occur at 1291 cm^−1^ [82]. As seen from Figure 4, the new peak appears at 1289 cm^−1^.

The C–H out-of-plane bending of 1249 cm^−1^ wavenumber in PAN 1 disappears in PAN 2. As a result, changes in shear and intensity are observed in PAN 2 when PVP is added. In general, while there is a shift to a lower number of waves, the signals intensify [83].

To note some key points among the results of the experimental study, PAN 1 has a modulus of elasticity of 66.31 MPa, with yield and tensile strength of 2.02 MPa and 3.63 MPa, respectively, while PAN 2 is much compliant and much weaker. PAN 2 has an elastic modulus of 21.89 MPa. Its yield and tensile strength lie at around 410 kPa and 1.38 MPa, respectively. Both of the materials have approximately the same Poisson ratio of 0.42. Furthermore, SEM analysis also supports these results by showing the internal structure of PAN 1 membranes with lower porosity. The internal structure of PAN 1 leads to stiffer behaviour, with higher yield and tensile strength compared to PAN 2 [69].

It is important to note that this polymer is usually reinforced with fibres or with supporting layers/laminae as their strength does not make them capable of being used alone.

It is also observed that the porosity, viscosity, water content, and surface roughness (RMS) are to be correlated to the stiffness and strength of the material. The reasoning for this correlation can be elucidated by commenting on the superficial mechanical behaviour of the microstructure and some particular material characteristics. Porous polymers are expected to have lower stiffness and less strength as pores cause stress concentrations as well as local plastic deformations. This is why stiffness and strength decrease as porosity increases. A very similar relationship can be found between the surface roughness, stiffness, and strength. The rougher the surface, the more stress concentrations are to be observed which cause more localised plastic behaviour. Furthermore, the increasing viscosity of the polymer has a negative effect on its stiffness and strength.

Lastly, PVP addition has a wide range of effects on material properties. As it is well known, PVP is added to increase the porosity of the membranes. Adding PVP to PAN increases the porosity as well as the surface roughness (RMS, consequently as explained earlier), PWF, viscosity, and water content but decreases the stiffness, strength, and contact angle. Similar results with different polymeric materials can be found in the literature [69].

### 4.2. Numerical Study: Modelling of Membranes

#### 4.2.1. Investigation of Membranes Made of PAN 1

In Table 3, the results of the finite element analysis, which is conducted for the membranes made of PAN 1, are presented. Analyses are conducted for different shapes, namely square, hexagon, circle, the rectangle with an aspect ratio of 2, and ellipses with different aspect ratios. The circular membrane is the one where the largest values of all the criteria are obtained. Circular is followed by the hexagon, square, and the rectangle with the aspect ratio of 2. A detailed investigation is conducted on elliptical membranes with aspect ratios from 1 to 4. A circle is an ellipse with an aspect ratio of 1.

According to the equivalent stress criteria of von Mises, the ellipse with the highest aspect ratio is the shape that could carry the highest pressure among the selected shapes. This conclusion is drawn from the fact that the lowest stress is observed on this membrane geometry. The best shape according to the equivalent strain and the displacement criteria is also the elliptical membrane with the aspect ratio of 4.

Another important aspect is the comparison of circular, hexagonal, and square shapes. These geometries have the same aspect ratio but they differ in their circumferential length over area ratio. The largest displacement, stress, and, consecutively, strain are observed in the circular membrane, which had the lowest circumference over the area ratio, although, the elliptical and the rectangular membranes had relatively close stress levels, with relatively smooth stress distributions. These mechanical quantities decreased as this ratio increased. This could be seen by investigating the change in the quantities in hexagonal and square shapes alongside the circular membrane. Square had the lowest values, whereas the hexagonal membrane fell in between circular and square. Square had the highest ratio and the circular had the lowest. It can be noted from the data presented in Table 3 that with an increasing circumference over area ratio, the reaction forces decrease. The same load is carried by the supports at the circumference. However, the longer the circumference, the less is the reaction. This can be explained by the different load transfers from the elastic membrane to the supports. The moment values at the supports are so low that it is possible to describe them as “practically zero” based on the very low bending stiffness of the membrane. The low bending stiffness of the membrane does not allow significant moment loads to coexist with forces at the supports as reactions.

**Figure 5:**
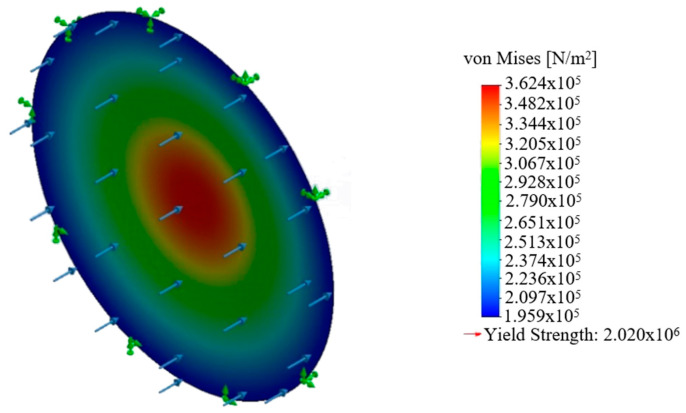
Equivalent stress distribution in a circular membrane.

Figure 5 represents the equivalent stress field in the circular membrane modelled using the finite element method. It can be seen that the stress distribution is smooth and there is no region where there is very low stress, whereas there are regions with very low stress in the square and hexagonal membranes. As the length of the circumference increased, the regions with low stress became wider, with high-stress regions and the maximum stress values decreasing as well.

**Figure 6:**
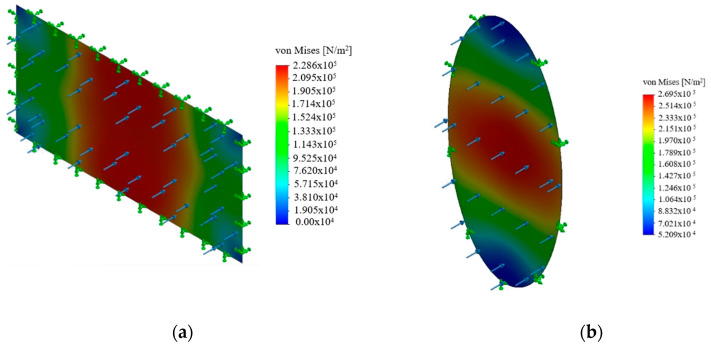
Equivalent stress distribution in (**a**) rectangular and (**b**) elliptical membranes with the aspect ratio of 2.

Figure 6 shows the von Mises stress distributions of membranes with elliptical and rectangular geometries that have the same aspect ratio of 2. As the circumference over area ratio increased, it could be seen that the regions where the stresses are small enlarged and the maximum stress value decreased. It could be seen that the area with small stress occupied a greater portion of the rectangular membrane and the stress distribution is more uniform in the elliptical membrane. The more uniformly distributed stress is more desired; hence, it reduced the maximum stress levels occurring in the membrane. The load is carried by the larger portion of the membrane. Considering applicability, both could be used in treatment systems. For example, for a membrane module, a rectangular membrane is more applicable due to its easier manufacturability. However, if the membrane should be located in a circular pipe, an elliptical is more likely to be applied since, with a certain angle, an elliptical membrane can be easily placed in a circular pipe.

#### 4.2.2. Investigation of Membranes Made of PAN 2

In Table 4, the results of the finite element modelling studies that are executed for the membranes made of PAN 2 are given. Based on these results, the best shape is determined as the elliptical geometry with the aspect ratio of 4. This geometry showed the lowest equivalent stress and strain along with the lowest resultant displacement. This allowed the membrane to carry higher amounts of pressure than the other selected geometries. Additionally, the stress distribution for this membrane is relatively smooth. This prevents local stress concentrations and may delay the failure under time-dependent loads and deformations.

As can be seen from Table 4, the resultant displacement and strain values for PAN 2 are considerably higher than those given in Table 3. The reason behind this is that the modulus of elasticity of PAN 1 is greater and the nonlinear behaviour of the materials is different. As the material is compliant and ductile, unlike PAN 1, greater strain values appear in PAN 2 and they are located at the supports. It can also be stated that relatively small strain values emerge in the circular membrane while membranes such as rectangles and ellipses, whose edges are at different distances from the midpoint, show the greatest strain values in the middle of the long edges. As the perimeter length and area ratios of the investigated shapes increase, it could be seen that the support reactions decrease. For membranes made of PAN 2, the support reaction moments are almost zero due to the very small bending stiffness of the membranes, as mentioned before.

The same comments which have been made for PAN 1 membranes on aspect ratio and circumference over area ratios can be made also for PAN 2 membranes. The comparison case is almost the same as PAN 1, with different magnitudes of these comparative quantities. As mentioned above for the case of PAN 1, elliptical membranes could be placed in a circular pipe with a certain angle between the normal surface of the membrane and the flow direction.

Lastly, PAN 1 and PAN 2 have significant differences in their mechanical behaviours. Considering the chemical/molecular structures of the polymer, of which both PAN 1 and PAN 2 membranes are made, are the same; the dominant factor that mainly influences the mechanical behaviour of these materials is judged to be the porosity. PAN1 with lower porosity is stiffer and stronger, whereas the more porous PAN 2 is more compliant and weaker. The presence of pores reduces the stiffness and leads to stress concentrations, which make it easy for plastic deformations to occur, cracks to initialise, and the membrane to fail.

#### 4.2.3. Investigation of Elliptical Membranes with Different Aspect Ratios

**Figure 7:**
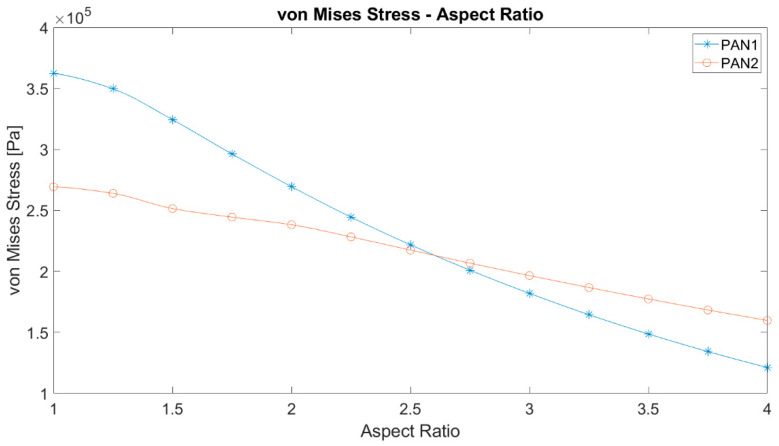
The maximum von Mises equivalent stress against the aspect ratio of elliptic membranes.

The equivalent stresses on PAN 1 and PAN 2 membranes with different aspect ratios are given in Figure 7. Based on the numerical studies performed, it can be claimed that the results for the two materials PAN 1 and PAN 2 might not have the same slope in stress criteria, which arises from the expectation of case dependency of such systems. However, a common observation is that the maximum stress decreases with rising aspect ratio. The greatest strain value in PAN 2 changes more significantly with the aspect ratio. The maximum stresses of PAN 1 and PAN 2 membranes equalise around the aspect ratio of 2.5 and 2.75.

**Figure 8:**
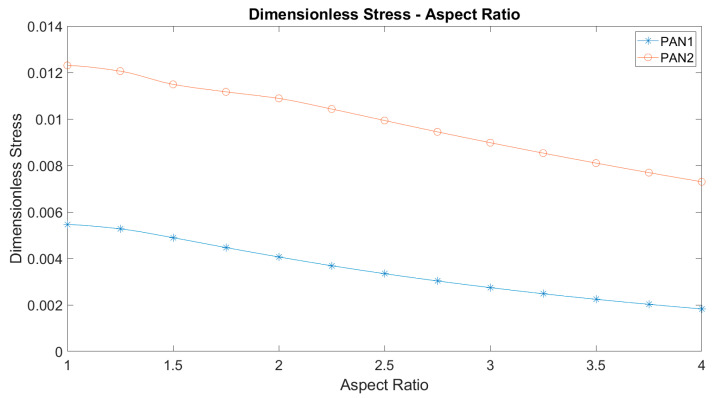
The maximum dimensionless stress against the aspect ratio of elliptic membranes.

Considering that the membrane models all remain well below the yielding point, a dimensionless analysis can be made by dividing the maximum von Mises stress by Young’s modulus. Figure 8 represents the dimensionless stress versus aspect ratio. The plots of so defined dimensionless stress versus the aspect ratio display a significant change in characteristics compared to the von Mises stresses. Both membrane materials show the same decreasing trend against aspect ratio and the curves do not intersect as a result, unlike von Mises stresses. In other words, the two curves are shifted. The shift can be explained with the only source of nonlinearity, which is the large deflections of the membranes. Therefore, the dimensionless stress magnitudes are different, similar to their stiffnesses.

**Figure 9:**
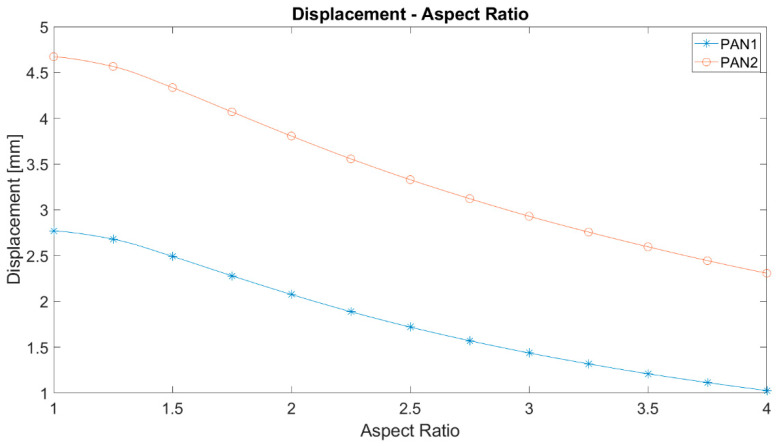
The maximum displacements against the aspect ratio of elliptic membranes.

As can be seen in Figure 9, the maximum displacement curves of PAN 1 and PAN 2 membranes versus aspect ratio have a negative slope as well. The higher the aspect ratio, the lower are the maximum displacements, but they have similar trends. Here, they only differ in their magnitudes as a consequence of their stiffness. Since PAN 2 is more compliant then PAN 1, PAN 2 deforms more than PAN 1. Thus, a stronger nonlinear characteristic is observed in PAN 2 membranes.

**Figure 10:**
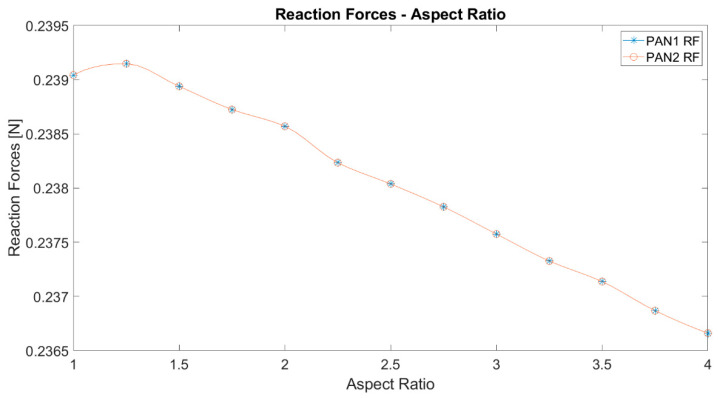
The total reaction force of elliptical membranes against the aspect ratio.

Another significant parameter to be investigated, as represented in Figure 10, is the total reaction forces at the supports. It can be noted that the total reaction forces decrease with increasing aspect ratio. This can be explained by the displacements. The initial load is perpendicular to the undeformed membrane surface. As the deformation occurs, the membrane surface deforms. As expected, the total reaction forces are the same for both types of membranes. Total reaction force has a maximum around the aspect ratio of 1.25.

#### 4.2.4. Final Remarks

Finally, the results show that the geometry does not mean only the form of the boundaries of membranes. It also includes the aspect ratio of the membrane, which is found to be as important as the curvature of the boundaries. It is expected that the sharp corners in the circumference would cause stress concentrations. From the figures above, it can be understood that the aspect ratio affects the stress, strain distribution, and deformations dramatically. This can be explained by the very close results of the circular and square membranes as well as the elliptic and the rectangular ones. Alongside the aspect ratio, the length of the circumference and area ratio are also influential on the mechanical behaviour of an elastic polymeric membrane. As the length increases, the resultant reaction forces reduce as well as the maximum stresses. This can be explained by the length of the circumference of the ellipses. Since the load applied is constant, the longer is the circumference, the less is the magnitude of the distributed line load. This leads to better loading and stress distribution all over the membrane. The equivalent strain has a maximum in the middle of the PAN 1 membrane, while the maximum occurs in the middle of the support in the long side for the PAN 2 membrane.

### 4.3. Numerical Study: Modelling of Representative Elements

**Figure 11:**
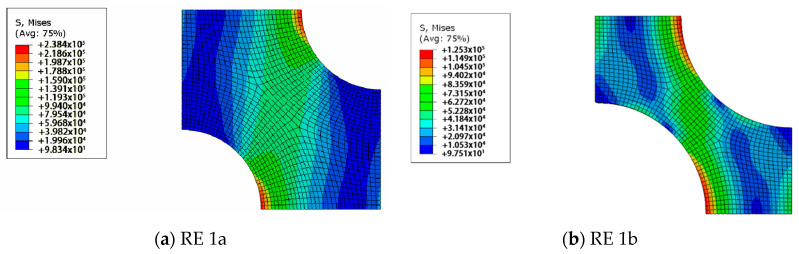
The von Mises stress distributions of RE 1.

**Figure 12:**
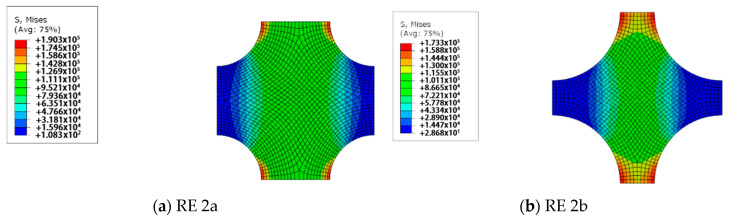
The von Mises stress distributions of RE 2.

Using the measured porosity values of PAN 1 and PAN 2 membranes, which are 23% and 51%, respectively, two-dimensional REs are prepared. First, the prepared four REs are analysed using the finite element method, as described earlier. After some iterations, taking PAN 1 as the reference for 23% porosity, Young’s modulus for the bulk material is estimated as 100 MPa. The results of the preliminary analysis with the calibrated modulus of elasticity are presented in Figure 11 and Figure 12.

For PAN 1, the RE with hexagonal pore array is called RE 1a which has 23% porosity. RE 1b, which represents PAN 2 only differs from RE 1a at its porosity value which is 51%. Similarly, RE with rectangular pore arrays with 23% and 51% porosities are referred to as RE 2a and 2b respectively, where RE 2a is generated to model PAN 1 and RE 2b for PAN 2.

Based on the results displayed in Figure 11, the effective modulus of elasticity for the RE with 51% (RE 1b) porosity is calculated as 24 MPa. With an approximate error of 12%, the estimation capability of RE 1 is acceptably accurate for this case. The error can be explained with other factors that are disregarded when building this model, such as irregular positioning and size distribution of pores and as well as surface roughness. Under the predefined strain, the maximum von Mises stress values of RE 1a and RE 1b are determined as 238 kPa and 125 kPa, respectively. Predictably, the stiffer structure displays greater stress under the same imposed displacement/strain. However, it does not suggest that the stiffer material would fail more easily than the more compliant material since, to give the same level of deformation to both the stiffer and the more compliant materials, stiffer material requires higher loads. It can also be claimed that there are high-stress regions from the equator of one pore towards the equator of the other pore. This high-stress region lies with a certain angle in the direction of tension. The existence and the position of this region can be explained by the intensified shear stresses around these areas. This region is more intense but smaller in RE 1b than RE 1a. This points out that the main load is carried by a smaller region in RE 1b than RE 1a, which leads to earlier failure even though the lower porosity values lead to smaller pores under the same distribution layout, which increases the stress concentrations around the pores.

As given in Figure 12, the results of RE 2 show that the general behaviour of RE 2 is considerably different compared to RE 1. First, the effective modulus of elasticity is obtained as 38 MPa for RE 2b. This is much more inaccurate compared to RE 1. Therefore, RE 1 is chosen as the better RE and it is used for further stages of the numerical study.

Regarding the results of RE 2, the maximum von Mises stresses in RE 2a and RE 2b are calculated as 193 kPa and 173 kPa, respectively. Similar comments can be made regarding their stiffness–stress relation, as done earlier for RE 1. The stress concentration controls the maximum stress value which occurs at the equators of each pore, as in RE 1. This is dominated by the size of the pore. On one hand, there are high-stress regions that peak at the equators of the pores, as in RE 1; on the other hand, the levels of stress are much lower and the high-stress regions, which lie parallel to the direction of tension, are spread over larger areas. In RE 1, the positioning of the pores reduces the shear stresses significantly. However, the stress concentrations at the equators of the pores are still noticeably high. Hence, the stress levels are significantly lower than the levels in RE 1. Similarly, RE 2b is expected to fail under a lower load than RE 2a because of the same reasons described for RE 1a and RE 1b.

Based on the results of the analysis with the REs, the better type of RE is determined as RE 1. Therefore, 14 new REs are generated and subjected to the same numerical analysis. As criteria to compare the results, the principal stresses and von Mises stress are chosen. The three values of principal stresses are read from the point where the domain maxima for the maximum principal stress is detected. Similarly, the von Mises stress values are read from the point where their domain maxima are located.

**Figure 13:**
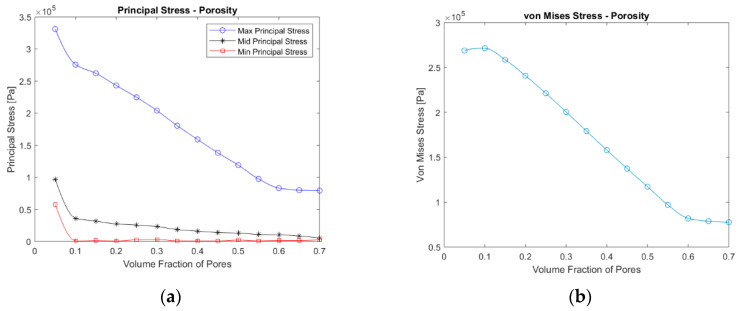
Von Mises (**a**) and principal (**b**) stress values in RE 1 under 0.001 uniaxial strain against porosity.

The results of this analysis are plotted in Figure 13. The maximum principal stress values display the exact same trend. They decrease monotonically with increasing porosity and seem to converge to a finite value after around 60%, whereas the maximum von Mises Stress values reach a maximum at around 10%. Then, they fall and finally converge to a finite value after around 60% of porosity, following a very similar trend as the principal stresses. One thing to mention about the magnitudes of the maximum principal and von Mises stress is that they have similar values. However, the maximum principal stress is found to have a shift which is almost 15% greater than the values of the von Mises stresses. Thus, the maximum principal stress criterion can be considered more conservative than von Mises. However, both equivalent stress concepts can be important based on the material and the chosen failure criteria.

## 5. Conclusions

In this study, the material and shape dependence of membrane systems are investigated. To obtain the necessary understanding of the material, uniaxial tensile testing is applied several times on several specimens. Following the experiment, numerical analyses are performed using finite element analysis. The analyses are constructed to compare the mechanical behaviour of different membrane geometries of the same surface area under the same loads and boundary conditions. The equivalent stresses and strains and displacements are calculated for each membrane. In addition, the resultant reaction forces and moments are obtained.

In the shape of circles and hexagons with edges which could be counted at equal distance from the centre, the stress values are relatively high, around 360 kPa and 260 kPa for PAN 1 and PAN 2, respectively. Besides this, the stress distributions in these membranes are smoother. However, there are regions where the stress values are small. As the aspect ratio increased, the greatest value of the stress decreased; regions with small stresses emerged, but regions with greater stresses grew.

It is seen that the maximum values of the stresses decreased as the aspect ratio of the ellipse increased. This reduction is found to be greater in PAN 2. It is obtained that the maximum equivalent stresses in PAN 1 and PAN 2 membranes are the same (around 200 kPa) between the aspect ratios of 2.5 and 2.75. The dimensionless stress is obtained by dividing the stress by Young’s modulus and it is plotted with aspect ratio. It is found that the curves obtained for PAN 1 and PAN 2 are of the same character and did not intersect each other. The dimensionless stress reduced almost linearly by 0.004 from the aspect ratios 1 to 4. The curve that belonged to PAN 2 is shifted upwards by 0.006. This difference in dimensionless stresses can be explained by the nonlinear deflections of the two membranes.

The results of numerical studies showed that the membrane systems should be considered as case-dependent, like many other nonlinear systems. The dependencies are dominated by the geometry and the material properties, as in this study. The reason behind this is the large displacements of the membranes under applied pressure. Therefore, for each type of material and each type of application, the analysis should be performed to obtain the best shape for the desired membrane system. After evaluating the results, it is concluded that alongside the geometry and the curvature of the boundaries, the aspect ratio of the membranes is also a dominant factor of the mechanical state of the membranes.

According to the results of the RE analysis, it is clearly shown that the hexagonal pore distribution, with six times less error, is more representative than the rectangular layout. The prediction accuracy for the elastic properties of the membrane is found to be satisfactory. Therefore, one of the dominant factors in the determination of the elastic properties can be named as porosity in such membrane materials. Comparing the equivalent stress criteria, it is shown that the maximum principal stress is more conservative than von Mises, as already known, but both of these follow quite parallel trends, with a difference of approximately 15%.

The workflow that is defined and described in this study gives an idea of how the membrane module, that is made of the chosen material, should be designed. Performing these numerical simulations simplifies the necessary experimentation process as some significant design parameters are determined through these simulations. This saves a good amount of time, resources, and cost as less experiments are required to design the membrane system.

## Figures and Tables

**Table 1:** The composition of PAN 1 and PAN 2 membranes.

Materials	Polymer Content %	PVP Content %	Solvent Content %
PAN 1	15	-	85
PAN 2	10	8	82

**Table 2:** Material properties.

	PAN 1	PAN 2
Material model type	Elastoplastic Isotropic	Elastoplastic Isotropic
Default failure criterion	Max von Mises Stress	Max von Mises Stress
Yield strength (MPa)	2.02	0.41
Tensile strength (MPa)	3.63	1.38
Elastic modulus (MPa)	66.31	21.89
Poisson’s ratio	0.42	0.42
Shear modulus (MPa)	23.35	7.77
Porosity (%)	23	51
Water content (%)	86	90
Contact angle (°)	49 ± 4.1	19 ± 4.3
Thickness (mm)	1.5	1.5
Viscosity (Pa.s)	1.9	2.8
Roughness RMS (nm)	15.4 ± 0.7	34.3 ± 2.5
PWF (Lm^−2^ h^−1^)	51.79 ± 2.59	91.48 ± 6.08

**Table 3:** Analysis results for PAN 1 membrane.

	von Mises Stress (N/m^2^)	Resultant Displacement (mm)	Equivalent Strain	Resultant Reaction Force (N)	Resultant Moment (N.m)
**Hexagon**	352,900	2.708	0.004799	0.237998	3.82465 × 10^−6^
**Circle**	362,400	2.771	0.00491	0.239043	5.59367 × 10^−7^
**Square**	329,300	2.551	0.004463	0.236367	7.15237 × 10^−6^
**Rectangle 2**	228,600	1.726	0.003099	0.237506	6.56278 × 10^−6^
**Ellipse 1.25**	349,700	2.68	0.004739	0.239148	2.99675 × 10^−6^
**Ellipse 1.5**	324,400	2.491	0.004392	0.238939	2.04139 × 10^−6^
**Ellipse 1.75**	296,300	2.279	0.004012	0.238725	6.54296 × 10^−7^
**Ellipse 2**	269,500	2.075	0.003649	0.238571	1.16314 × 10^−6^
**Ellipse 2.25**	244,500	1.887	0.003312	0.238234	7.11306 × 10^−7^
**Ellipse 2.5**	221,800	1.719	0.003005	0.238038	2.07609 × 10^−7^
**Ellipse 2.75**	201,000	1.57	0.002723	0.237827	3.75788 × 10^−7^
**Ellipse 3**	182,000	1.436	0.002466	0.237576	7.83426 × 10^−6^
**Ellipse 3.25**	164,500	1.317	0.00223	0.237327	2.73056 × 10^−7^
**Ellipse 3.5**	148,700	1.209	0.002015	0.237137	7.21739 × 10^−7^
**Ellipse 3.75**	134,300	1.113	0.00182	0.236869	5.21311 × 10^−7^
**Ellipse 4**	121,200	1.025	0.001642	0.236661	1.43855 × 10^−7^

**Table 4:** Analysis results for PAN 2 membrane.

	von Mises Stress (N/m^2^)	Resultant Displacement (mm)	Equivalent Stain	Resultant Reaction Force (N)	Resultant Moment (N.m)
Hexagon	265,100	4.572	0.05629	0.237998	3.79577 × 10^−6^
Circle	269,400	4.673	0.05715	0.239043	5.65258 × 10^−7^
Square	254,700	4.394	0.06405	0.236367	7.14436 × 10^−6^
Rectangular 2	227,900	3.355	0.06261	0.245	6.56139 × 10^−6^
Ellipse 1.25	264,000	4.566	0.05507	0.239148	2.97877 × 10^−6^
Ellipse 1.5	251,500	4.335	0.06064	0.238939	2.03055 × 10^−6^
Ellipse 1.75	244,500	4.07	0.06237	0.238726	6.52721 × 10^−7^
Ellipse 2	238,300	3.806	0.06048	0.238572	1.16243 × 10^−6^
Ellipse 2.25	228,300	3.557	0.05719	0.238235	7.09091 × 10^−7^
Ellipse 2.5	217,500	3.329	0.05444	0.238039	2.05879 × 10^−7^
Ellipse 2.75	206,800	3.122	0.05114	0.237828	3.75808 × 10^−7^
Ellipse 3	196,600	2.93	0.04748	0.237576	7.83368 × 10^−7^
Ellipse 3.25	186,800	2.757	0.04373	0.237326	2.7316 × 10^−7^
Ellipse 3.5	177,400	2.596	0.03991	0.237136	7.21877 × 10^−7^
Ellipse 3.75	168,400	2.445	0.0361	0.236868	5.20782 × 10^−7^
Ellipse 4	159,800	2.307	0.03345	0.23661	1.43446 × 10^−7^
